# Protease nexin-1 expression is altered in human breast cancer

**DOI:** 10.1186/1475-2867-6-16

**Published:** 2006-05-31

**Authors:** Britny J Candia, William C Hines, Christopher M Heaphy, Jeffrey K Griffith, Robert A Orlando

**Affiliations:** 1 Department of Biochemistry and Molecular Biology, University of NewMexico, School of Medicine, MSC08 4670, 1 University of New Mexico, Albuquerque, New Mexico, 87131, USA

## Abstract

**Background:**

Urokinase-type Plasminogen Activator (uPA), a serine protease, plays a pivotal role in human breast cancer metastasis by mediating the degradation of extracellular matrix proteins and promoting cell motility. In more advanced breast cancers, uPA activity is significantly up regulated and serves as a prognostic indicator of poor patient outcome. Classically, regulation of uPA activity, especially in breast cancers, is thought to be mediated by Type 1 Plasminogen Activator Inhibitor (PAI-1). However, we have recently found that a lesser known natural inhibitor of uPA, Protease Nexin 1 (PN-1), is expressed in normal human mammary tissue. Based on this observation, we investigated if PN-1 is also expressed in human breast cancers where it may contribute to the regulation of uPA and participate in the development of a metastatic phenotype.

**Results:**

Using quantitative real-time PCR analysis, we measured PN-1 mRNA expression in tissues obtained from 26 human breast tumor biopsies and compared these values with those obtained from 10 normal breast tissue samples. Since both PAI-1 and uPA expression levels are known to be elevated in metastatic breast cancer, we also measured their levels in our 26 tumor samples for direct comparison with PN-1 expression. We found that PN-1 expression was elevated over that found in normal mammary tissue; an increase of 1.5- to 3.5-fold in 21 of 26 human breast tumors examined. As anticipated, both PAI-1 and uPA mRNA levels were significantly higher in the majority of breast tumors; 19 of 26 tumors for PAI-1 and 22 of 26 tumors for uPA. A quantile box plot of these data demonstrates that the elevated PN-1 expression in breast tumor tissues directly correlates with the increased expression levels found for PAI-1 and uPA.

**Conclusion:**

The fact that PN-1 expression is elevated in human breast cancer, and that its increased expression is directly correlated with increases measured for PAI-1 and uPA, suggests that PN-1 may contribute to the regulation of uPA-mediate tumor cell motility and metastatic spread.

## Background

An important characteristic of highly invasive tumor cells is an elevated capacity to degrade the surrounding extracellular matrix (ECM). To achieve this elevated degradative capacity, tumor cells express a variety of proteases to digest ECM proteins that typically encapsulate growing, benign tumors [1,2]. It is now well established that proteins of the plasminogen activation (PA) system are elevated in breast cancer and serve as the primary functional players in ECM degradation [3,4]. Expression of one member of the PA system, the serine protease urokinase (uPA), is significantly upregulated in tumor cells and catalyzes the conversion of extracellular plasminogen to plasmin [5]. Plasmin is a broad-spectrum protease that cleaves many ECM proteins, as well as activates certain matrix metalloproteinases [6]. This proteolytic cascade enables highly migratory tumor cells to efficiently degrade their surrounding matrices, exit the primary site of tumor growth and colonize distant secondary sites [7]. In addition to its protease activity which augments breast tumor cell motility, high expression levels of uPA is also a well-established prognostic indicator of poor patient outcome during the course of breast cancer [8,9].

Regulation of extracellular uPA activity is known to occur through the inhibitory properties of type I plasminogen activator inhibitor (PAI-1), a serine protease inhibitor (SERPIN) that is synthesized and secreted often by the same cells that secrete uPA [10]. Because of the close functional relationship between uPA proteolytic activity and PAI-1 inhibitory function, it is thought that a well-controlled balance of uPA and PAI-1 dictates the extent of cell motility. Protease Nexin-1 (PN-1), another member of the SERPIN family [11], is highly expressed by stromal cells [12] and a potent inhibitor of uPA [10,13]. Interestingly, although PN-1 activity has been extensively studied within the context of neural development, few studies have been reported examining its expression in cancerous tissues and its potential role in cancer progression. PN-1 is expressed by astrocytes and glial cells [14], as well as neuroblastoma cells [15] where it is thought to promote neuronal cell survival [16] and modulate neurite outgrowth [17]. In addition, PN-1 inhibits thrombin-stimulated cell division [18], migration of cerebrellar granular cells [19], and uPA-dependent ECM degradation [20]. Thus, based on findings in other cell types, we hypothesize that PN-1 may contribute to tumor cell motility in advanced stage breast cancer by playing a role in the regulation of uPA activity. To address this hypothesis, we examined the expression of PN-1 in advanced stage human breast cancer tissues to determine if its expression is altered when compared to normal mammary tissue and to directly compare its expression level to those of PAI-1 and uPA. To accomplish this goal, we used quantitative real-time reverse transcription-PCR (QRT-PCR) to measure PN-1, PAI-1 and uPA expression levels within a set of breast tumor and normal breast tissue samples.

## Materials and methods

### Breast tissue samples

Frozen breast tumor specimens from anonymous patients (n = 26) were obtained from the University of New Mexico Cancer Research and Treatment Center Solid Tumor Facility, Albuquerque, New Mexico. In 25 out of 26 cases, tumor grade, tumor size, lymph node status, and the fraction of cells in S phase (based on flow cytometry cell cycle analysis) were included within the clinical history provided with each specimen. Anonymous normal breast mRNA (n = 10) originating from female patients where cause of death was unrelated to cancer, were purchased from Ambion (Austin, TX). The normal, control samples were supplied as two equal pools by the company.

### Cell culture

MCF-7 human mammary epithelial cells, were provided by Dr. Steven Abcouwer, Hershey Medical Center, Hershey, Pennsylvania. MDA-MB-231 metastatic human mammary epithelial cells were obtained from American Type Culture Collection (Rockland, MD). Both cell lines were propagated in Dulbecco modified Eagle's medium (DMEM, Life Technologies/Invitrogen, Carlsbad, CA) supplemented with 10% fetal calf serum (Irvine Scientific, Santa Ana, CA), 0.1 mM non-essential amino acids, 1 mM sodium pyruvate, 0.01 mg/ml bovine insulin, and 100 U/ml penicillin G. Cells were cultured at 37°C with 5% CO_2 _and passaged once a week.

### Preparation of tissue sections and RNA isolation

Serial frozen sections of breast samples, 10 μm in width, were mounted on Colorfrost slides (VWR, West Chester, PA) and stored at -70°C. Specimens were stained with hematoxylin/eosin and examined by a board-certified surgical pathologist, who assigned a histopathologic grade to the tumor and analyzed the normal tissue. Total RNA from cultured cells and frozen tumor tissue was isolated using silica-based spin-column extraction kits (RNeasy/DNeasy mini kits, Qiagen, Valencia, CA) according to the manufacturer's protocol. Total RNA was treated with RNase-free DNase I (Ambion, Austin, TX) to eliminate contaminating DNA. RNA integrity was evaluated by agarose gel electrophoresis.

### Quantitative real-time RT-PCR

cDNA was synthesized by random decamer-primed reverse transcription of RNA (1 μg) using a TaqMan^® ^Reverse Transcription kit (Applied Biosystems, Foster City, CA) according to the manufacturer's standard protocol. Negative controls contained RNase-free water substituted for reverse transcriptase. The mRNA levels of PN-1, PAI-1, uPA and TATA-binding protein (TBP) were measured in breast specimens, the MCF-7 mammary epithelial cell line, and the MDA-MB-231 metastatic mammary epithelial cell line using the ABgene Absolute SYBR Green QRT-PCR assay (Fisher Scientific, Hampton, NH). PN-1 primers were selected to amplify an 81 bp sequence spanning the intron located between exons 2 and 3. Primer sequences used for PN-1 were 5'-GAAGCAGCTCGCCATGGT-3' (forward), 5'-AGACGATGGCCTTGTTGATC-3' (reverse). TBP primer sequences used were 5'-CACGAACCACGGCACTGATT-3' (forward), 5'-TTTTCTTGCTGCCAGTCTGGAC-3' (reverse). Primer sequences used for PAI-1 were 5'-TGCTGGTGAATGCCCTCTACT-3' (forward), 5'-CGG TCA TTC CCA GGT TCT CTA-3' (reverse). uPA primer sequences used were 5'-CAC GCA AGG GGA GAT GAA-3' (forward), 5'-CA GCA TTT TGG TGG TGA CTT-3' (reverse) [21]. Final concentration of PN-1, PAI-1 and uPA primers used for amplification was 600 nmol/L forward, 600 nmol/L reverse; 600 nmol/L forward, 900 nmol/L reverse was used for TBP primers. Amplification of PN-1, PAI-1, uPA and TBP cDNA was performed using the MiniOpticon Real-Time PCR Detection System (Biorad, Hercules, CA). The cycling parameters used were as follows: 1 cycle, 95°C for 10 min; 50 cycles, 95°C for 15 sec and 60°C for 1 min; 1 cycle, 40°C for 3 min. The PN-1, PAI-1 and uPA mRNA levels were normalized to TBP mRNA levels using the Comparative C_T _method and are reported in the figures as fold difference compared to levels found in normal mammary tissue. Melting curve analyses were performed for all amplifications to verify that only single products were generated from the reactions. Amplicons were sequenced to verify authentic PN-1. The cDNA for human PN-1 was obtained from the I.M.A.G.E. Consortium (ID: 4824856; Genbank: BC042628; Genbank: BC042628).

## Results

### Quantitation of PN-1 expression in human breast tumors

For QRT-PCR analysis, we designed primers to amplify an 81 bp sequence of PN-1 spanning the splice junction between exons 2 and 3. Spanning a splice junction ensures that amplified products are derived solely from mRNA and not from genomic DNA that might remain in our preparation. In order to test the specificity of these novel primers, we amplified the 81 bp PN-I sequence by straight RT-PCR using RNA purified from a normal human fibroblast cell line (HuFb) and compared the product to that obtained from amplification using the human PN-1 cDNA. We chose to use human fibroblasts since they synthesize and secrete active PN-1 at levels corresponding to ~1% of all secreted proteins [22,23]. As anticipated, we found that our newly designed primers amplified only the expected 81 bp sequence (data not shown).

**Figure 1:**
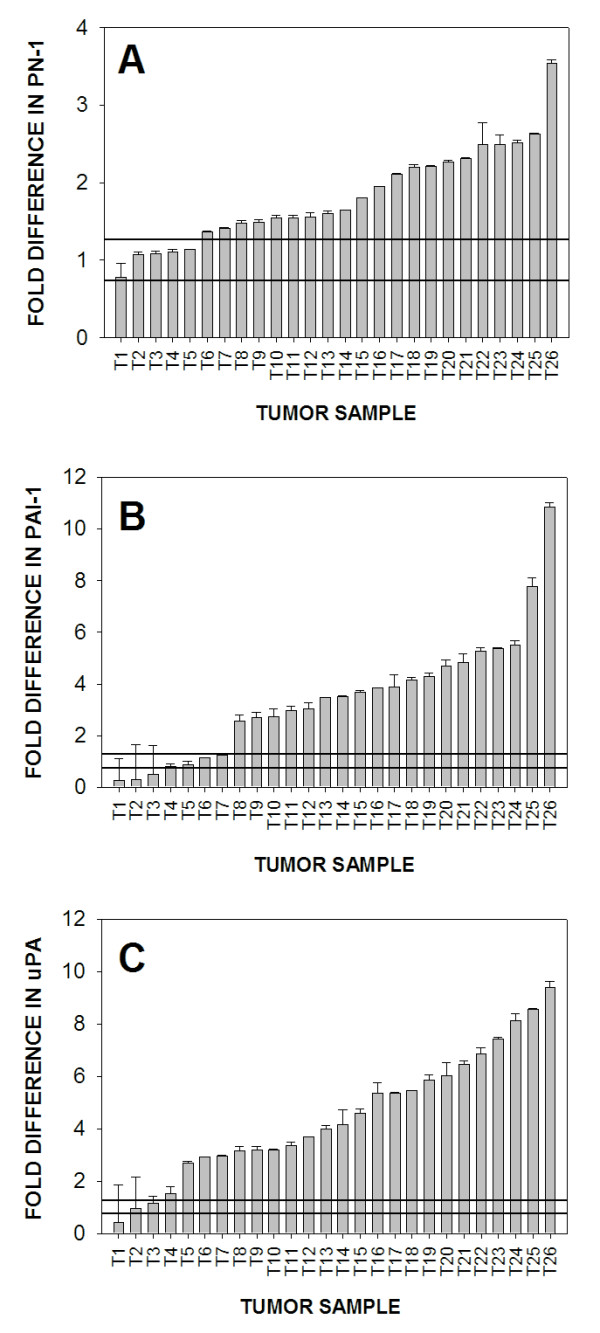
**PN-1, PAI-1 and uPA mRNA expression in human breast tumor tissues and normal human mammary tissue**. RNA was isolated from 26 breast tumors (T1–T26) and 10 normal breast samples. Normal samples were pooled into two equal groups (N1 and N2). mRNA levels for each gene were evaluated by QRT-PCR. Relative levels of PN-1, PAI-1 and uPA mRNA were normalized to TATA binding protein mRNA levels. Comparative C_T _method was used to calculate fold difference of PN-1 (A), PAI-1 (B) and uPA (C) expression in breast tumor tissue as compared to levels measured in normal breast tissue. The mean value of the two pooled normal samples was calculated and assigned a value of one in order to determine relative fold change of expression within the tumor samples. The standard deviation of the normal samples was 0.263. The box represents one standard deviation of the mean values obtained from normal mammary tissues.

**Figure 2:**
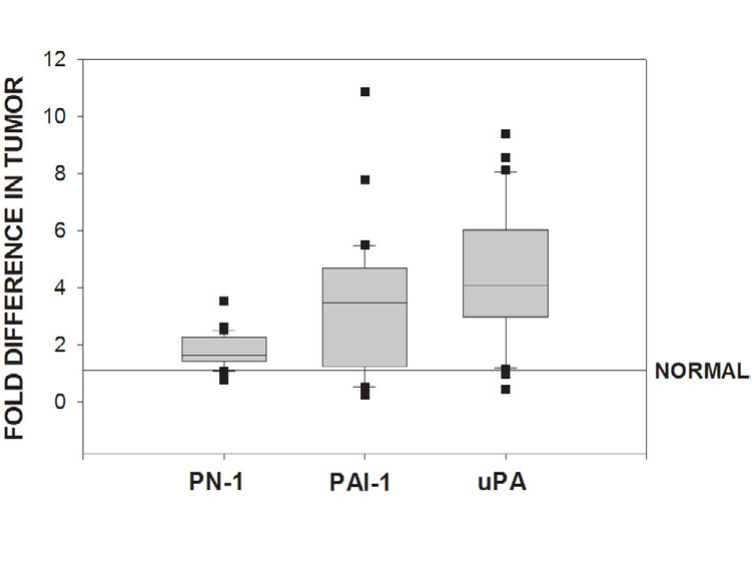
**Comparison of PN-1, PAI-1 and uPA mRNA expression levels in 26 breast tumor samples**. The box for each gene represents the interquartile range (25–75th percentile) and the line within this box is the median value. Bottom and top bars of the whisker indicate the 10th and 90th percentiles, respectively. Outlier values are indicated (closed squares).

To quantify PN-1 expression in human breast cancers, we obtained 26 samples of breast tumor tissue, purified RNA and generated cDNA from this material. The cDNA were then analyzed by quantitative PCR and the results were compared directly to quantitative PCR values obtained using cDNA generated from normal human mammary tissue. We found that PN-1 expression was elevated over that found in normal mammary tissue; an increase of 1.5- to 3.5-fold in 21 of 26 human breast tumors examined (Fig. 1A). Since both PAI-1 [24-26] and uPA [24,26] expression levels are known to be elevated in metastatic breast cancer, we measured their levels in our 26 tumor samples for direct comparison with PN-1 expression. As anticipated, both PAI-1 (Fig. 1B) and uPA (Fig. 1C) mRNA levels were significantly higher in the majority of breast tumors; 19 of 26 tumors for PAI-1 and 22 of 26 tumors for uPA. A quantile box plot of the data shown in Figure 1 permits a direct comparison of expression levels for PN-1, PAI-1 and uPA (Fig. 2). These data clearly show that PN-1 expression is elevated in the majority of human breast cancers examined and that this elevated expression directly correlates with the expected higher expression levels found for PAI-1 and uPA. Since the majority of our tumor samples represent advanced stage, grade 2 and 3 breast cancers, we are unable to determine at this time if PN-1 expression levels correlate with tumor grade, lymph node status or patient reoccurrence.

### PN-1 expression in MCF-7 and MDA-MB-231 breast cancer cells

**Figure 3:**
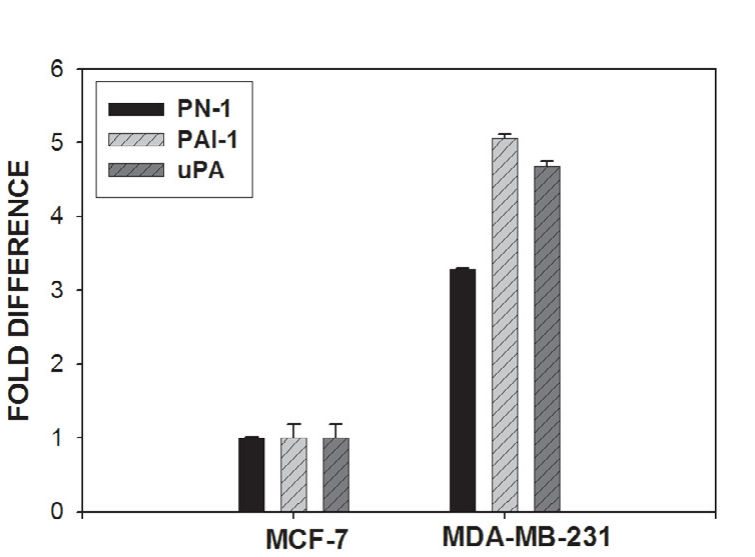
**QRT-PCR analysis of PN-1, PAI-1 and uPA mRNA expression in MCF-7 and MDA-MB-231 breast cancer cell lines**. PN-1, PAI-1 and uPA message levels were quantitated in MCF-7 and MDA-MB-231 cells by QRT-PCR. Expression levels for PN-1, PAI-1 and uPA were normalized to values obtained for TATA binding protein. C_T _values for each gene obtained from MCF-7 cells were averaged and assigned a value of one to assess relative fold increase in expression in MDA-MB-231 cells.

We plan to characterize the mechanism responsible for increased expression of PN-1 in breast cancer and determine its functional role in breast cancer metastasis. To accomplish this goal, we will require a cultured model system for accurate, controlled assessment of PN-1 promoter status, transcription factor requirements, and tumor cell invasive capacity. MCF-7 and MDA-MB-231 cells are well established cultured lines used extensively to study molecular details of breast cancer progression [27]. Hormone-responsive MCF-7 cells have a low invasive capacity and represent earlier stages of breast cancer, while hormone-independent MDA-MB-231 cells are highly invasive and represent more advanced stage breast cancer. To determine if these cell models will be useful for examining PN-1 function in breast cancer, we performed QRT-PCR analysis to identify if PN-1 expression is disregulated in a comparable manner to that seen in human breast cancer tissues. We found that MDA-MB-231 cells express 3.5-fold greater levels of PN-1 than MCF-7 cells (Fig. 3). Moreover, increased expression of PAI-1 and uPA were also found in MDA-MB-231 cells as compared to MCF-7.

## Discussion

Advanced stage breast cancer is accompanied by a dramatic increase in metastatic potential of epithelial-derived tumor cells. The observed increase in tumor cell motility is aided by increased expression and activity of uPA [4]. For effective tumor cell migration, the proteolytic activity of uPA is thought to be balanced by the inhibitory activity of PAI-1 [28,29]. The cycling activities of proteolysis and protease inhibition lead to sequential rounds of cell detachment-reattachment, which in turn leads to an increase in cell motility. Indeed, elevated expression levels of both uPA and PAI-1 are characteristic of advanced stage breast cancers [30]. Interestingly, although PN-1 is structurally and functionally related to PAI-1, there have been no studies to date investigating if PN-1 contributes to breast cancer progression in a manner similar to that of PAI-1. To address this gap in knowledge, we examined if PN-1 expression is altered in human breast cancer by quantitating levels of PN-1 expression in human tissue samples obtained from tumor biopsies. In these same samples, we also quantitated PAI-1 and uPA expression levels for direct comparison to PN-1. Our findings indicate that PN-1 expression is elevated in the majority of human breast tumor tissues examined and that its expression levels are directly correlated with increases measured for PAI-1 and uPA. We also found that the highly metastatic, MDA-MB-231 breast cancer line expresses 3.5-fold greater levels of PN-1 compared to the non-tumorgenic, MCF-7 breast cancer cell line. This increase in PN-1 is also correlated to increases seen for PAI-1 and uPA in MDA-MB-231 cells. The elevated expression of all three genes is consistent with our measurements in human breast tumor samples. The significant differences in PN-1 expression between non-tumorigenic MCF-7 cells and highly invasive MDA-MB-231 cells should provide us with a good basis for identifying the mechanism responsible for altered PN-1 expression seen in breast tissues and allow us to examine PN-1 function in the context of elevated PAI-1 and uPA levels. Taken together, these data indicate that PN-1 expression is increased during breast cancer tumorigenesis and may contribute, along with PAI-1, to uPA-mediated tumor cell motility and a more advanced metastatic phenotype.

Although studies in the literature investigating a role for PN-1 in cancer progression are limited, our results complement and extend data presented in a recent report by Buchholz and colleagues [31]. Their study demonstrated that a highly metastatic pancreatic cancer line overexpressed PN-1, while a less metastatic subclone showed little PN-1 expression. The authors also noted that stable PN-1 overexpression in the less metastatic subclone greatly enhanced its local invasiveness in *in vivo *studies. Our studies expand on these observations by demonstrating an increase in PN-1 expression in human breast cancer tissues.

Quantitating expression of PAI-1 and uPA is of high prognostic value for assessing breast cancer survival outcome [24,25]. Numerous independent studies have shown that patients with low levels of PAI-1 and uPA in their primary tumor tissue have a significantly better survival rate than patients with high levels of either factor alone. Recently, the prognostic value of PAI-1 and uPA has been verified by a pooled analysis consisting of >8,000 breast cancer patients [26]. In light of the overlapping protease specificities of PAI-1 and PN-1 [32], together with the established role of PN-1 in neuronal cell regulation and motility [33,34], we believe it is likely that PN-1 also plays a role in breast cancer progression by contributing to events necessary for increased tumor cell motility. Increased expression of PN-1 by tumor cells may serve to modulate their adhesiveness or motility [35]. Alternatively, tumor cell activity may be influenced by tumor-stromal tissue crosstalk [36]. The breast neoplastic stroma contains a heterogeneous cell population composed of fibroblasts, myofibroblasts, and endothelial cells, which are all known to synthesize and secrete significant amounts of PN-1 [37]. Although it remains to be determined the precise mechanism by which PN-1 contributes to breast cancer tumor progression, the results of the present study establish a rationale for further investigation of PN-1 as a modulator of uPA activity in breast tumor cell motility. Future studies will be focused on identifying transcriptional and/or translational mechanisms controlling PN-1 expression by cancer cells and determining if PN-1 serves as an independent prognostic indicator of breast cancer staging by using a more widely defined sample of tumor tissues, including earlier stage cancers as well as late-stage carcinomas. In addition, the use of laser capture dissection technology will confine our QRT-PCR measurements to tumor tissue and eliminate contributions from surrounding normal mammary tissue that are likely to occur when using surgical specimens.

## Conclusion

We quantitated PN-1 expression in samples obtained from biopsies of human breast tumors and from normal mammary tissues by QRT-PCR analysis and compared these results to those obtained for PAI-1 and uPA. Our findings indicate that PN-1 expression is elevated in a majority of human breast tumor tissues examined when compared to normal human mammary tissue. In addition, the elevated PN-1 expression in tumor tissues directly correlates with increased expression measured for PAI-1 and uPA. We also found that the highly metastatic, MDA-MB-231 breast cancer cell line expresses greater levels of PN-1 compared to the non-tumorgenic, MCF-7 breast cancer cell line. Consistent with observations obtained from tumor biopsies, PN-1 expression levels in MDA-MB-231 directly correlate with increases found for PAI-1 and uPA. These data indicate that PN-1 expression is increased during breast cancer tumorigenesis and may contribute, along with PAI-1, to uPA-mediated tumor cell motility and a more advanced metastatic phenotype.

## Abbreviations

uPA, urokinase-type plasminogen activator; PAI-1, plasminogen activator inhibitor; ECM, extracellular matrix; PA, plasminogen activation; SERPIN, serine protease inhibitor; PN-1, protease nexin-1.

## Competing interests

The author(s) declare that they have no competing interests.

## Authors' contributions

BJC carried out the majority of studies and drafted the manuscript. WCH and CMH provided confirmed breast tumor and normal mammary samples. JKG assisted with data interpretation. RAO provided the original conceptual framework for the study, participated in the experimental design and finalized the manuscript for submission. Authors read and approved the final version.
